# Pain During a Marathon Run: Prevalence and Correlates in a Cross-Sectional Study of 1,251 Recreational Runners in 251 Marathons

**DOI:** 10.3389/fspor.2021.630584

**Published:** 2021-02-10

**Authors:** Patrick J. O'Connor

**Affiliations:** Department of Kinesiology, University of Georgia, Athens, GA, United States

**Keywords:** exercise, pain intensity, pain threshold, perceived exertion, running, sex differences

## Abstract

This cross-sectional study aimed to obtain fundamental knowledge about pain during a marathon run. During the first seven months of 2007, announcements on websites of interest to marathon runners were used to recruit participants. A total of 1,251 runners (550 female runners) completed a 41-question online survey about the location and intensity of their primary pain during their last marathon and potentially related variables [perception of effort during the marathon, number of prior marathons run, typical pain intensity during training runs, percentage of training days with run-induced pain, highest intensity pain ever experienced]. Pain location was selected from a list of 27 specified body sites covering the entire body. Kilometer at which pain first occurred indexed pain threshold. Pain intensity at the primary location of pain was measured with a standardized, well-validated 0–10 pain intensity scale. Pearson correlations and multiple regression quantified the associations between average pain intensity and other variables. Sex-related differences in pain were tested using independent *t*-tests. Effort ratings (6–20) were added as a covariate in an ANCOVA to test if perceived effort accounted for possible sex-related differences in pain. Based on the available research, it was hypothesized that: (i) most runners would report moderate intensity pain, (ii) pain would be associated with both exercise intensity during the marathon and pain during training, and (iii) after adjusting for expected sex-related differences in perceived effort, females would experience pain earlier and rate the pain intensity as higher. All but two runners (99.8%) reported pain during a marathon, and most frequently in the anterior/medial thigh (17.1%), hamstring (10%), and calf (9.3%) locations. Pain threshold occurred at 25.3 ± 9.8 km (15.7 ± 6.1 miles) and the overall pain intensity of the run was 5.26 ± 2.45. No sex-related pain differences were found. Overall pain intensity during a marathon was significantly associated with: pain intensity during training runs (*r* = 0.39), percentage of training days with run-induced pain (*r* = 0.23), highest intensity pain ever experienced (*r* = 0.23), number of prior marathons (*r* = −0.18), and intensity of effort (r = 0.11) (all *P* < 0.001). Most runners experience moderate to very strong intensity pain during a marathon; the pain was independent of biological sex, and the pain is weakly associated with marathon race experience, pain during training, race effort, and the highest intensity of pain ever experienced.

## Introduction

Completing a marathon run is difficult and includes multiple challenges including coping with the naturally occurring pain experienced during the exercise (Cook et al., 1997). This paper focuses on naturally occurring pain during running and not on pain caused by a major musculoskeletal injury or pain that is common after a marathon that is often delayed in onset by 24–72 h. Pain has been defined by experts as an unpleasant sensory and emotional experience associated with, or resembling that associated with, actual or potential tissue damage (IASP, 2012). Pain experts also have concluded that “pain is always a personal experience” and that “a person's report of pain should be respected.” One highly cited study found that elite male distance runners used elaborate cognitive association and dissociation techniques to attempt to better tolerate pain during marathon races (Morgan and Pollack, 1977). This early work was conducted with elite athletes. However, non-elite runners account for the majority of the approximately 1.1 million runners who participate in organized marathon races worldwide each year (Anderson, 2019).

Better understanding pain during a marathon is potentially important for several reasons. One reason is the paucity of empirical information about pain during a marathon among non-elite distance runners. Related studies have examined pain in association with a single marathon race, using a small sample size and pain was assessed after the exercise. Thus, the location, intensity, and prevalence of pain during a marathon is uncertain and an initial estimate could be obtained from a cross-sectional study. A second reason is to discover relationships between pain during a marathon and other important related outcomes. For example, a marathon often results in delayed onset muscle injury but the extent to which this type of pain and pain during a marathon are related has never been explored. One recent investigation did find that pain data obtained immediately after a marathon run resulted in unique associations with biological outcomes compared to the commonly assessed delayed onset measures of pain that are obtained 1–3 days post-marathon (Tokinoya et al., 2020). Analogously, there is a need to know whether unique and useful insights are gleaned from obtaining pain data during a marathon and relating it to outcomes of interest such as those involved in recovery from a marathon run. A third type of reason includes potential relationships between pain and psychological factors such as self-identity (Lev, 2019), feelings of accomplishment (Hammer and Podlog, 2016), and enjoyment of distance running (Bale, 2006). A fourth type of reason for understanding pain during a marathon involves potential relationships with behavioral factors such as adherence to regular running (Slimmon et al., 2002) and performance in a marathon race (Stevens et al., 2018). While these relationships are plausible, fundamental questions related to pain during a marathon have not yet been addressed adequately by researchers. Questions of interest include the onset of pain during a marathon and its overall intensity.

If these questions are to be answered and our understanding of pain during marathon running is to advance more fully, it is critically important to obtain empirical data describing plausible correlates of pain during marathon running (Sparling et al., 1993; Curtis et al., 2016). For instance, perceptions of effort ratings are frequently obtained during exercise but only relatively recently has there been interest in obtaining concomitant pain ratings during exercise (Cook et al., 1997; Staiano et al., 2018). A notable exception has been the recording chest pain among those with a suspected or actual cardiac condition (O'Connor and Cook, 1999). So, correlational data are needed to better understand the overlap and distinctions between the related concepts of pain and effort perceptions. Also, prior pain experiences can influence subsequent pain responses to noxious stimuli (Rollman et al., 2004). Thus, it would be potentially important to generate empirical data examining whether substantial relationships exist between pain during a marathon run and experience with prior pain. Potentially important correlates include general pain experience measures such as of the most intense pain ever experienced (Roy et al., 1989). Other variables of interest are running specific measures such as the percentage of training days that running-induced pain occurred and the average intensity of pain at the primary pain location during training (Lohrer et al., 2018).

Closely related research has tested potential treatments for muscle pain experienced post-marathon (Shanely et al., 2014), documented pain sensitivity to cold or pressure following longer ultramarathon runs (Freund et al., 2013), or been focused on specific types of pain associated with marathon running such as breast pain (Brown et al., 2014). One study (*n* = 127) of primarily male runners (85% of the sample) reported a mean pain immediately after a marathon run that was of moderate intensity (~5 on a 0–10 scale). However, no assessment of pain intensity during the run was made (Babel et al., 2018).

In part because more research has been conducted with males than females historically, another question of substantial interest concerns potential sex-related differences. A wealth of literature has shown sex-related differences in pain (Bartley and Fillingim, 2013), including musculoskeletal pain (Rollman and Lautenbacher, 2001). Compared to groups of males, female samples are characterized by a lower pain threshold and report higher pain ratings when given standardized noxious stimuli in laboratory settings (Riley et al., 1998). If these lab-based findings generalized to pain during marathon running, they might help to explain other observations such as the sex-related difference in pacing during the marathon (Hubble and Zhao, 2016). Whether there are sex-related differences in pain during marathon running appears to have never been investigated.

To address some of the questions introduced above, a cross sectional survey study of recreational marathon runners was conducted in order to obtain some initial fundamental knowledge about pain during a marathon run. The aims of this cross-sectional study were: (i) to describe pain experiences during a 42.2 km (26.2 mile) marathon (i.e., primary body location of pain during exercise, its average intensity during the run, and when during the run the pain started), (ii) to quantify selected correlates of the average pain intensity experienced at the primary location of pain during a marathon, and (iii) to consider possible sex-related differences.

Based on the report of moderate mean pain intensity post-marathon (Babel et al., 2018), it was hypothesized that most participants would report pain during the marathon (>50%) and on average, the pain would be of moderate intensity. It was also hypothesized that the intensity of effort put into the run would be significantly correlated with the average pain intensity during the marathon based on experiments showing that perceived exertion ratings are positively related to pain intensity during other types of exercise (Cook et al., 1997). Based on the general psychological principal that past behavior guides future behavior (Ouellette and Wood, 1998) as well as evidence that this idea is supported by studies of physical activity behavior habits (Rebar et al., 2016), it was also hypothesized that average pain intensity during the marathon would be associated with the presence of pain experienced while training for the marathon. Lastly, it was hypothesized that females would experience pain earlier during the marathon and would rate the intensity as higher, even after adjusting for expected sex-related differences in effort (Garcin et al., 2005).

## Methods

### Design

A cross-sectional design was used to answer the research questions. Adult participants were recruited using online announcements that asked marathon runners to complete a survey of ~10 min. All methods were approved by the Institutional Review Board prior to collecting data and written informed consent was obtained from all participants (University of Georgia IRB, #200701014).

### Participant Recruitment

A cross-sectional online survey was used, rather than studying runners from a single, local marathon race, to obtain information from a broader sample of marathons and runners in order to increase the generalizability of the findings. Announcements were available from January 1, 2007 to July 31, 2007 on websites of interest to marathon runners including race websites (i.e., Chicago, Georgia, Grandma's, Twin Cities), a running shoe and apparel website https://www.runningwarehouse.com), an online magazine (Runner's World), an online discussion group (LetsRun), and emails sent to individual runners known to the investigator. No other media was used to disseminate the information. The announcements had a link to the survey questions, which were administered electronically via Survey Monkey (www.surveymonkey.com).

### Survey

The survey was developed for the study by the author (PJO) with assistance from a research assistant (JD), both of whom had marathon running experience. The author drafted the questions and the research assistant reviewed the items and suggested edits for clarity. After this, the survey was administered to three local runners who were asked for clarity suggestions. The final survey consisted of 41 questions. The final survey is available in the Supplementary Appendix.

The primary outcomes were pain location, pain threshold, and pain intensity. The primary location of pain during the most recent marathon was selected from a list of 27 specified body sites covering the entire body. A 28th option allowed participants to choose, but not specify, “some other site.” The location categories were based on a widely used body figure pain drawing classification system (cf. O'Connor et al., 2000) that was adapted by the investigator for running. The adaptation involved both increasing the number of lower leg categories to obtain more specific information on pain sites (e.g., adding lateral and medial knee locations) and combining some upper body locations expected to have infrequent pain (e.g., arm pain was used rather than separate reports of left and right lower arm, elbow, and upper arm sites). The International Association for the Study of Pain defines pain threshold as “The minimum intensity of a stimulus that is perceived as painful.” The term pain threshold, however, is also widely used to describe when a non-painful stimuli, such as pressure applied to the finger or heat or cold applied to the skin, becomes painful as the duration of exposure to the stimuli continues. An analogous pain threshold measure was used in the present investigation. Pain threshold was inferred from the following question, which did not specify pain location, “At what mile did you begin to feel pain during your last marathon?” The average intensity of pain during the marathon at the primary pain location was queried using a 0–10 numerical category pain intensity scale that includes the anchors: 1 = weak pain, 2 = mild pain, 3 = moderate pain, 5 = strong pain, and 7 = very strong pain. Results from numerous studies support the validity of this scale as a measure of pain intensity (Cook et al., 1997). The validity evidence includes data obtained during moderate intensity cycling and running exercise modes with durations of 30 to more than 60 min (Lopez et al., 2011; Herring et al., 2019).

Other outcomes included finishing time and the overall perceived intensity of effort put into the most recent marathon run was reported using Borg's 6–20 scale (Borg, 1998). There is substantial evidence to support the validity of this approach to measuring perceived exertion across an entire exercise session (Haddad et al., 2017). A subset of questions also asked about responses to training which preceded the most recent marathon run and were thought to be of potential relevance to pain experienced during the marathon. These included an estimate of the percentage of training days that running-induced pain occurred and the average intensity of pain at the primary pain location during training.

### Statistical Analysis

Data were imported into the Statistical Package for Social Sciences (version 24) for analysis. Respondents who provided inaccurate answers to questions that could be verified (e.g., responses outside the possible range on a variable, such as age >120) were excluded. Pairwise deletion was used to manage missing data. Significance was set to *P* < 0.001, rather than 0.05, to conservatively minimize potential bias resulting from conducting multiple statistical tests.

#### Prevalence Data

Means and 95% confidence intervals described the pain intensity data, frequency of occurrence described the body location, and both frequency as well as mean and 95% confidence intervals described the pain threshold data.

#### Correlational Data

Pearson correlations, and associated 95% confidence intervals, quantified the associations between average pain intensity experienced at the primary location of pain and (i) pain intensity felt during training runs, (ii) the percentage of training days with run-induced pain, (iii) the highest intensity pain ever experienced, (iv) the number of prior marathons run, and (v) relative exercise intensity as indexed by perception of effort. Partial correlation coefficients were used to examine whether these relationships were influenced by the number of weeks since the last marathon was run. Multiple regression examined the relationship between average pain intensity experienced at the primary location of pain as the dependent variable and the set of independent variables above (i–v) as well as performance time and biological sex.

#### Sex-Differences

Mean differences in pain between sexes was assessed using independent *t*-tests and effect sizes were calculated using Cohen's d. Differences in the location of pain between sexes was assessed with z-scores for tests of two proportions. Effort ratings were added as a covariate in an ANCOVA to test if effort accounted for possible sex-related differences in pain intensity.

For all three categories of data, sensitivity analyses were conducted to consider if the findings were influenced when those reporting pain at the starting line (*n* = 147 participants) were removed from the data set.

## Results

Data from 19 respondents were excluded for inaccurate answers to questions that could be verified. There was a total of 1.4% of missing data among the total 51,291 responses to the 41 question survey. Descriptive statistics for the female and male samples, including sample size variations because of missing data across the variables, are provided in Table 1. The participants ran in 252 different marathons held in Australia, North America, and Europe, most (95%) were run in the United States. The median and mode number of weeks since participating in the target marathon was 21 and 1, respectively.

**Table 1:** Characteristics of the female and male samples of marathon runners surveyed.

**Variable**	**Sex**	**N**	**Mean**	**SD**	**Cohen's d**	**Sex difference *p*-value**
Age-years	Female	552	36.03	10.15	0.40	<0.001
	Male	696	40.32	11.22		
Body mass-kg	Female	535	59.65	7.54	1.73	<0.001
	Male	688	75.08	10.10		
Pain at primary pain location 0–10	Female	553	5.43	2.45	0.13	0.026
	Male	698	5.12	2.43		
Highest intensity pain ever experienced 0–10	Female	551	9.11	2.60	0.05	0.353
	Male	695	9.24	2.66		
Prior marathons run	Female	553	6.84	12.46	0.15	0.009
	Male	698	8.78	13.47		
% of training days with running-induced pain	Female	553	24.44	23.20	0.15	0.008
	Male	698	21.02	21.97		
Pain intensity at primary location in training 0–10	Female	553	4.97	1.89	0.21	<0.001
	Male	698	4.56	1.95		
Perceived exertion 6–20	Female	553	14.38	2.18	0.28	<0.001
	Male	698	14.99	2.24		
42K run time minutes	Female	523	261.95	49.99	0.63	<0.001
	Male	665	230.58	49.67		
Start line pain intensity 0–10	Female	553	1.40	1.15	0.07	0.239
	Male	698	1.32	1.07		

### Prevalence

**Figure 1:**
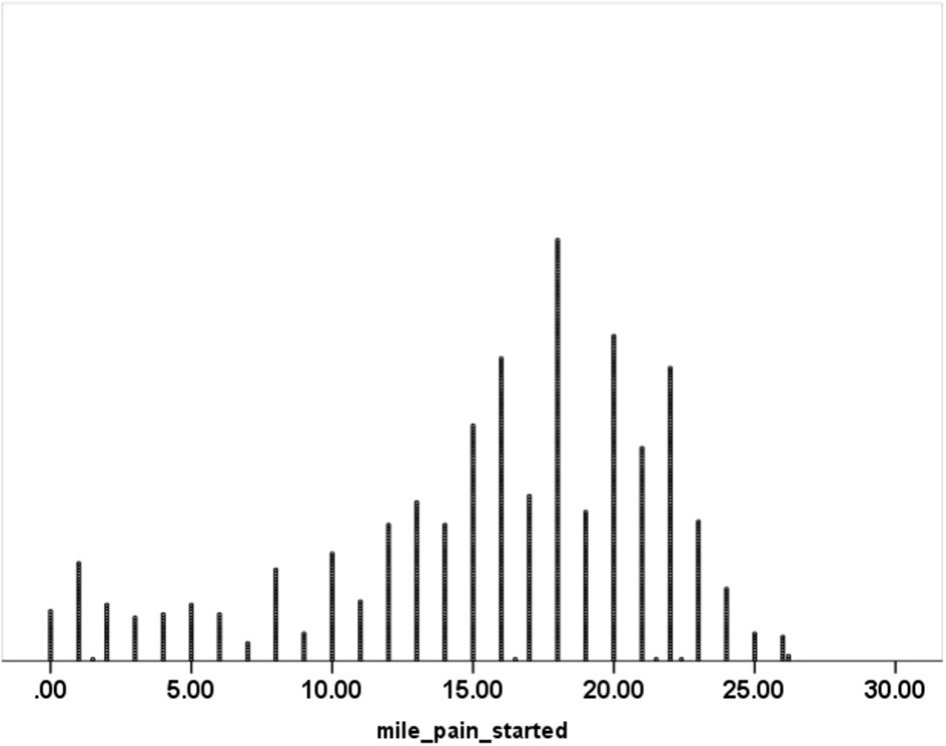
Frequency distribution of the mile at which pain was first experienced during a marathon.

Pain threshold was normally distributed and on average occurred at 25.3 ± 9.8 km (15.7 ± 6.1 miles) into the run (95% CI: 24.6–25.9 km and 15.3–16.1 miles). These data are illustrated in Figure 1. Similar results were observed after those reporting pain at the starting line were removed in a sensitivity analysis: 25.9 ± 9.0 km (16.1 ±5.6 miles).

The mean and SD pain intensity at the primary location were 5.26 and 2.45 while the 95% confidence interval ranged from 5.12 to 5.40. The mean and SD pain intensity at the primary location were 5.14 and 2.44 after those reporting pain at the starting line were removed in a sensitivity analysis.

The primary sites that pain was experienced most frequently during the marathon were the anterior/medial thigh (17.0%), hamstring (10.1%), and calf (9.3%) muscle groups. The data for all the body locations are presented in Table 2.

**Table 2:** Body locations of primary pain during a marathon run.

**Location of primary pain during 42k run**	**Percentage of the sample reporting**
Anterior/medial thigh	17.0%
Hamstring	10.1%
Calf	9.3%
Anterior knee	5.2%
Lateral knee	5.0%
Iliotibial band	4.6%
Ball of foot	4.1%
Hip	3.3%
Hip flexor	2.9%
Low back	2.7%
Toe	2.4%
Arch of foot	2.3%
Medial knee	2.2%
Heel of foot	2.1%
Ankle	1.8%
Abdominals	1.6%
Buttocks	1.6%
Shins	1.5%
Top of foot	1.4%
Groin	1.3%
Chest	0.7%
Shoulders	0.7%
Upper back	0.3%
Neck	0.3%
Arms, hands or fingers	0.2%
Head	0.1%
Some unspecified other location	2.8%
No primary location	10.2%

### Correlations

The bivariate correlation results are presented in Table 3.

**Table 3:** Correlations and 95% confidence intervals for average pain intensity and pain threshold.

	**Pain intensity**		**Pain threshold**	
**Variable**	**r-value**	**95% CI**	**r-value**	**95% CI**
Pain intensity during training runs	0.39^*^	0.35–0.44	−0.22^*^	0.16–0.28
Training days with run-induced pain	0.23^*^	0.18–0.28	−0.32^*^	0.27–0.37
Highest intensity pain ever	0.23^*^	0.17–0.28	0.05	−0.01 to 0.10
Number of prior marathons run	−0.18^*^	0.12–0.22	0.01	−0.06 to 0.08
Relative exercise intensity	0.11^*^	0.05–0.16	0.05	−0.01 to 0.11
Relative exercise intensity controlling for marathon run time	0.18^*^	0.12–0.23	−0.01	−0.05 to 0.07

* *P < 0.001*.

Statistically significant bivariate correlations were found that were weak in magnitude between average pain intensity during the marathon and measures of prior pain experience, marathon running experience, and relative exercise intensity.

The multiple regression showed that each of the independent variables contributed significantly to the prediction of average pain intensity during the marathon (*R* = 0.473; *R*^2^ = 0.224; *P* < 0.001). The standardized beta coefficient (β) and significance for each variable was: (i) pain intensity felt during training runs (0.300; *p* < 0.001), (ii) the highest intensity pain ever experienced (0.168; *p* < 0.001), (iii) total run time (0.128, *p* < .001), (iv) perceived exertion (0.120, *p* < 0.001), (v) the number of prior marathons run (−0.109; *p* < 0.001), and (v) percentage training days with running-induced pain (0.090, *p* = 0.001).

Statistically significant bivariate correlations that were weak in magnitude were observed between pain threshold and pain during training.

The significance and strength of these bivariate associations did not change in partial correlation analyses that statistically accounted for the number of weeks since the last marathon was run or the presence/absence of pain at the starting line.

### Sex Differences

There was not a significant difference between the female and male sample for the number of weeks since the last marathon had been completed. The percentage of female and male samples reporting pain did not differ significantly at any of the 27 body sites tested. The mean ± SD km (mile) at which the females and males began to feel pain during the marathon were 24.6 ± 9.8 and 25.7 ± 9.8 km (15.3 ± 6.1 and 16.0 ± 6.1 mile), respectively. The associated 95% confidence intervals for the female (23.7–25.4 km and 14.7–15.8 mile) and male (24.7–26.4 km and 15.5–16.4 miles) samples overlapped and there was no significant sex difference in pain threshold (*t* = 1.877, *df* = 1,109, *p* = 0.061; *d* = 0.11). The average pain intensity was non-significantly higher for the female (5.43 ± 2.45) vs. the male (5.12 ± 2.43) sample and the difference did not become significant after accounting for sex-related differences in effort or the absence/presence of pain at the starting line in an ANCOVA. Biological sex was not a statistically significant predictor of average pain intensity at the primary location of pain when it was added to the multiple regression (standardized beta = 0.11; *p* = 0.684).

## Discussion

The primary findings of this investigation include that essentially everyone experiences pain during a marathon (99.8%). For most individuals, the pain during a marathon run begins between 24 and 26 km (mile 15 and 16), most frequently in the anterior/medial thigh, hamstring, and calf muscle groups. This pain for most runners on average is moderate-to-strong in intensity. This type of acute pain is independent of biological sex, and tends to be worse among those reporting more pain during training, less marathon experience, higher effort, and higher intensity of the worst pain ever experienced.

### Pain Threshold

On average, runners reported pain starting between 24 and 26 km (mile 15 and 16) of the marathon. However, there was substantial variability in the onset of pain during a marathon, ranging from within the first km/mile to the last km/mile of the race. Substantial inter-individual variability characterizes pain threshold measures even when standardized noxious stimuli are presented to a homogeneous group in a lab setting, including cycling exercise stimuli (Cook et al., 1997; Cathcart and Pritchard, 2006). Thus, it was not surprising that there was substantial variability in the km/mile at which pain was first experienced during a marathon run. Plausible related to this was that the absolute exercise stimulus was not the same for every participant because of variations in, for example, race course elevation. Despite the variability, it was logical that runners who reported a lower pain intensity during training and a lower percentage of training days with run-induced pain also tended to report a pain threshold that occurred later in their marathon run. These relationships were weak in magnitude and the lower bound of the 95% confidence interval did not overlap zero. A significant negative correlation between pain threshold and perceived exertion might have been expected logically but these two variables are known to be independent in some circumstances (Staiano et al., 2018).

### Pain Location

The fact that pain was experienced most frequently in the anterior/medial thigh, hamstring, and calf muscle groups is consistent with prior reports of hamstring and calf injuries among marathon runners (Satterthwaite et al., 1999). The hypothesis that most runners (>50%) would experience pain during the marathon was confirmed. Indeed, only 2 of 1,251 runners reported feeling no pain during a marathon. These data indirectly support the idea that pain is not a primary constraint on human endurance performance (Staiano et al., 2018). For example, ultramarathon runners can continue to endure for 160 km or more per day (100 miles) despite the presence of pain (Hoffman et al., 2007). It may be possible for a higher percentage of participants to run a pain-free marathon if they are well-trained for the task, and if a key goal is to run at a pace that avoids pain. It is unknown how many people who participate in a marathon, run with a primary goal to avoid pain. The motivations for running were not assessed among the participants in the present study. Individuals train for and run marathons for a host of reasons, including to lose weight, to increase physical fitness, to give life meaning, to help cope with life's problems, to socialize, to achieve a goal, and others (Masters and Ogles, 1998; Hammer and Podlog, 2016). Certain types of goal achievement motivations, such as running a fast time, are inconsistent with avoiding pain.

### Hypothesis of Moderate Intensity Pain for Most Runners

The hypothesis that most people would experience moderate intensity pain during the marathon was not confirmed. The average pain intensity at the primary location of pain represented pain that was “strong.” Strong pain is more intense than moderate on the scale used. The mean pain intensity was less than the highest intensity pain ever experienced, which in this study approached the extremely intense category of the 0–10 scale used. About two thirds of the sample reported overall pain that ranged from 3 (moderate) to 7 (very strong). The only prior study that measured the pain of running a marathon found that recalled pain intensity of the marathon 1 week or 1 month after the marathon averaged between 5 and 6 on an 11-point numerical scale (Babel et al., 2018). These observations are generally consistent with the present findings. The prior study manipulated the timing of the recall post-marathon and compared participants who were, and were not, experiencing pain during the recall period. The authors concluded that pain intensity experienced during a marathon run is underestimated 1 month after the marathon compared to a week after, and is mediated by the pain experienced when the recall is made (Babel et al., 2018). No direct comparison can be made between this prior smaller study and the present study because of methodological and study aim differences. In the current study, the presence of pain during the recall was not obtained and the time since the last marathon varied. In the present study, the time since the last marathon was measured. Removing the influence of that variable did not significantly change the strength of associations based on partial correlation analyses.

### Hypothesized Correlates of Pain Intensity During a Marathon

As hypothesized, the intensity of pain during training and the percentage of training days in pain were significantly and positively, though weakly, related to pain during the marathon. These relationships seem plausible in part because whatever caused the pain during training, such as an injury, hilly training routes, or high intensity effort, could have carried over to the race day. Prior aerobic training (Owen et al., 2019) and physical fitness (Schmitt et al., 2020) might have influenced pain during the marathon. Alternatively, non-running related variables could be important moderators, including genetics (Meloto et al., 2018), family history of hypertension (Cook et al., 2004), typical sleep duration (Herrero Babiloni et al., 2020), and health status.

It is unclear why the average pain intensity during the marathon was positively and weakly correlated with the highest intensity pain ever experienced. Speculation about masochistic and addictive elements of long distance running could be relevant (Nogueira et al., 2018). Also, rating judgements may be different after exposure to a high intensity pain if it resets the high-end anchor on the pain scale (Rollman et al., 2004). Future research is needed to fully understand this finding. Possible explanations for the weak negative correlation between the number of prior marathons and pain during the marathon appear more straightforward. Multiple psychophysiological adaptations from training for, and competing in, marathon runs serve to reduce pain and effort perceptions associated with the marathon (Sparling et al., 1993).

Effort ratings were weakly and positively correlated with the average pain intensity experienced during the marathon run (r = 0.11) and the magnitude of the relationship was higher after controlling for run time (*r*_12.3_ = 0.18). Pain and effort are conceptually positively related; exerting more effort results in running faster which increases the likelihood of higher intensity pain. The magnitude of these relationships, however, can be influenced by a host of variables. These variables include whether one is running uphill or downhill (Garnier et al., 2018), recent prior run training (Black and Dobson, 2013), exercise duration (Slapšinskaite et al., 2017), and health status (Cook et al., 2003). Most of the available data about pain and perceived effort during exercise comes from studies of indoor cycling. Thus, the present study adds novel data to the small literature on pain, effort, and marathon running.

### Hypothesized Sex-Related Differences

On average, females did report higher average pain intensity at the primary location of their pain sooner in the race than males by an average of 0.3 raw pain score units. The magnitude of this difference was not enough to support the hypothesized sex-related difference in this variable, even after adjusting for the sex-related difference in effort. The observation of lower effort ratings, on average, for the female sample compared to the males is consistent with prior studies. These studies show that females, compared to males often exercise at a lower intensity and engage in physical activity modes that are less strenuous (Mcarthur and Raedeke, 2009; Watson et al., 2015). In marathon running, however, women maintain a steady pace to a greater extent than men. This plausibly could contribute to lower effort ratings, though the reason for this sex-related difference is unknown (Deaner et al., 2015).

### Strengths, Weaknesses, and Implications for Research and Practice

Pain experienced during marathon running has received little research attention. This paper addressed part of this knowledge gap. A key strength of this study was the broad sample of marathon runners that included females and males, younger and older adults, and individuals who ran in 251 different marathon races across multiple continents.

This investigation had several weaknesses. Given the epidemiological nature of the study, it was not possible to conduct interviews with participants in order to clarify the nature of the stimulus for their pain. The presence of pain at the starting line among 147 participants, for example, may have been the result of a chronic injury and consequently their reports of pain may have differed from those who were not injured. When these individuals were removed in the sensitivity analyses, large differences yielding different conclusions did not emerge. The study also was not controlled in a way that each runner ran the same course. Consequently, error variance in estimating pain intensity during the marathon potentially was increased because of differences in the courses run. The survey was not a random sample from a well-defined population of marathon runners. Therefore, the findings may not generalize to all other samples of marathon runners. Nonetheless, the findings contribute to the literature because the substantial number of participants, the diversity of marathon courses, and the absence of similar data. Comparisons between females and males were imperfect because they were not based on samples carefully matched to avoid confounding variables such as the course run, prior training, running speed, and other variables that could have influenced pain intensity. The participants in this study recalled pain related to their last marathon, thus potential recall bias could have influenced the outcomes. The extent to which the present findings, based on data obtained in 2007, generalize to 2020 is uncertain given the potential for cohort effects associated with changes in environmental or cultural variables across time. Lastly, it was a limitation that numerous biological (e.g., skeletal muscle fiber type), psychological (e.g., whether an individual catastrophizes about pain), and social factors (e.g., running alone or with a partner) that can influence pain were not measured or accounted for in the present investigation.

The present results are of potential practical use to clinicians, coaches, and novice marathon runners because it gives them science-based information about the timing and magnitude of pain to be expected during a marathon run. Investigators can use the present findings as a platform to conducted future experimental trials that rigorously control variables of interest such as the marathon course and environment. The present data also may inform future studies that match men and women on training prior to the marathon in order to better understand pain during marathon running.

## Conclusion

The present study shows that a marathon run is associated with moderate to severe pain for most runners. This type of acute pain is independent of biological sex, and tends to be worse among those reporting more pain during training, less marathon experience, higher effort, and higher intensity of the worst pain ever experienced.

## Data Availability Statement

The raw data supporting the conclusions of this article will be made available by the authors, without undue reservation.

## Ethics Statement

The studies involving human participants were reviewed and approved by University of Georgia Institutional Review Board. The patients/participants provided their written informed consent to participate in this study.

## Author Contributions

PO planned and designed the study, analyzed the data, and wrote the manuscript.

## Conflict of Interest

The author declares that the research was conducted in the absence of any commercial or financial relationships that could be construed as a potential conflict of interest.

## Abbreviations

CI: confidence interval
SD: standard deviation.

## References

[B1] Anderson J. J. (2019). The State of Running. Available online at: https://www.iasp-pain.org/terminology?navItemNumber=576#Pain (accessed January 27, 2021).

[B2] Babel P. Bajcar E. A. Smieja M. Adamczyk W. Swider K. Kicman P.. (2018). Pain begets pain. When marathon runners are not in pain anymore, they underestimate their memory of marathon pain—-A mediation analysis. Eur. J. Pain 22, 800–809. 10.1002/ejp.1166 29271541

[B3] Bale J. (2006). “The place of pain in running,” in Pain and Injury in Sport: Social and Ethical Analysis, eds S. Loland, B. Skirstad, and I. Waddington (Routledge: New York, NY), 65–75.

[B4] Bartley E. J. Fillingim R. B. (2013). Sex differences in pain: a brief review of clinical and experimental findings. Br. J. Anaesth. 111, 52–58. 10.1093/bja/aet127 23794645 PMC3690315

[B5] Black C. D. Dobson R. M. (2013). Prior eccentric exercise augments muscle pain and perception of effort during cycling exercise. Clin. J. Pain 29, 443–449. 10.1097/AJP.0b013e318262ddfe 23328320

[B6] Borg G. (1998). Borg's Perceived Exertion and Pain Scales. Champaign, IL: Human Kinetics.

[B7] Brown N. White J. Brasher A. Scurr J. (2014). The experience of breast pain (mastalgia) in female runners of the 2012 London Marathon and its effect on exercise behaviour. Br. J. Sports Med. 48:320. 10.1136/bjsports-2013-092175 23603819

[B8] Cathcart S. Pritchard D. (2006). Reliability of pain threshold measurement in young adults. J. Headache Pain 7, 21–326. 10.1007/s10194-006-0265-7 16440140 PMC3451574

[B9] Cook D. O'connor P. Eubanks S. Smith J. Lee M. (1997). Naturally occurring muscle pain during exercise: assessment and experimental evidence. Med. Sci. Sports Exerc. 29, 999–1012. 10.1097/00005768-199708000-00004 9268956

[B10] Cook D. B. Jackson E. M. O'connor P. J Dishman R. K. (2004). Muscle pain during exercise in normotensive African American women: effect of parental hypertension history. J. Pain 5, 111–118. 10.1016/j.jpain.2003.12.002 15042519

[B11] Cook D. B. Nagelkirk P. R. Peckerman A. Poluri A. Lamanca J. J. Natelson B. H. (2003). Perceived exertion in fatiguing illness: civilians with chronic fatigue syndrome. Med. Sci. Sports Exerc. 35, 563–568. 10.1249/01.MSS.0000058360.61448.6C 12673137

[B12] Curtis E. A. Comiskey C. Depsey O. (2016). Importance and use of correlational research. Nurse Res. 6, 20–25. 10.7748/nr.2016.e1382 27424963

[B13] Deaner R. O. Carter R. E. Joyner M. J. Hunter S. K. (2015). Men are more likely than women to slow in the marathon. Med. Sci. Sports Exerc. 47, 607–616. 10.1249/MSS.0000000000000432 24983344 PMC4289124

[B14] Freund W. Weber F. Billich C. Birklein F. Breimhorst M. Schuetz U. H. (2013). Ultra-marathon runners are different: investigations into pain tolerance and personality traits of participants of the TransEurope FootRace 2009. Pain Pract. 13, 524–532. 10.1111/papr.12039 23368760

[B15] Garcin M. Fleury A. Mille-Hamard L. Billat V. (2005). Sex-related differences in ratings of perceived exertion and estimated time limit. Int. J. Sports Med. 26, 675–681. 10.1055/s-2004-830440 16158374

[B16] Garnier Y. M. Lepers R. Dubau Q. Pageaux B. Paizis C. (2018). Neuromuscular and perceptual responses to moderate-intensity incline, level and decline treadmill exercise. Eur. J. Appl. Physiol. 118, 2039–2053. 10.1007/s00421-018-3934-8 29992466

[B17] Haddad M. Stylianides G. Djaoui L. Dellal A. Chamari K. (2017). Session-RPE method for training load monitoring: validity, ecological usefulness, and influencing factors. Front. Neurosci. 11:612. 10.3389/fnins.2017.00612 29163016 PMC5673663

[B18] Hammer C. Podlog L. (2016). “Motivation and marathon running,” in Marathon Running: Physiology, Psychology, Nutrition and Training Aspects, eds C. Zinner, and B. Sperlich (Cham: Springer International Publishing), 107–124. 10.1007/978-3-319-29728-6_6

[B19] Herrero Babiloni A. De Koninck B. P. Beetz G. De Beaumont L. Martel M. O. Lavigne G. J. (2020). Sleep and pain: recent insights, mechanisms, and future directions in the investigation of this relationship. J. Neural Transm. 127, 647–660. 10.1007/s00702-019-02067-z 31452048

[B20] Herring M. P. Monroe D. C. Gordon B. R. Hallgren M. Campbell M. J. (2019). Acute exercise effects among young adults with analogue generalized anxiety disorder. Med. Sci. Sports Exerc. 51, 962–969. 10.1249/MSS.0000000000001860 30531490 PMC7218918

[B21] Hoffman M. D. Lee J. Zhao H. Tsodikov A. (2007). Pain perception after running a 100-mile ultramarathon. Arch. Phys. Med. Rehabil. 88, 1042–1048. 10.1016/j.apmr.2007.05.004 17678668

[B22] Hubble C. Zhao J. (2016). Gender differences in marathon pacing and performance prediction. J. Sports Analytics 2, 19–36. 10.3233/JSA-150008

[B23] IASP (2012). Classification of Chronic Pain, 2nd ed, Revised E-book. Available online at: http://www.iasp-pain.org/PublicationsNews/Content.aspx?ItemNumber=1673andnavItemNumber=677

[B24] Lev A. (2019). Becoming a long-distance runner – deriving pleasure and contentment in times of pain and bodily distress. Leis. Stud. 38, 790–803. 10.1080/02614367.2019.1640776

[B25] Lohrer H. Malliaropoulos N. Korakakis V. Padhiar N. (2018). Exercise-induced leg pain in athletes: diagnostic, assessment, and management strategies. Phys. Sportsmed. 47, 47–59. 10.1080/00913847.2018.1537861 30345867

[B26] Lopez R. M. Casa D. J. Jensen K. A. DeMartini J. K. Pagnotta K. D. Ruiz R. C.. (2011). Examining the influence of hydration status on physiological responses and running speed during trail running in the heat with controlled exercise intensity. J. Strength Cond. Res. 25, 2944–2954. 10.1519/JSC.0b013e318231a6c8 22024610

[B27] Masters K. S. Ogles B. M. (1998). The relations of cognitive strategies with injury, motivation, and performance among marathon runners: results from two studies. J. Appl. Sport Psychol. 10, 281–296. 10.1080/10413209808406394

[B28] Mcarthur L. H. Raedeke T. D. (2009). Race and sex differences in college student physical activity correlates. Am. J. Health Behav. 33, 80–90. 10.5993/AJHB.33.1.8 18844523

[B29] Meloto C. B. Benavides R. Lichtenwalter R. N. Wen X. Tugarinov N. Zorina-Lichtenwalter K.. (2018). Human pain genetics database: a resource dedicated to human pain genetics research. Pain 159, 749–763. 10.1097/j.pain.0000000000001135 29300278

[B30] Morgan W. P. Pollack M. (1977). Psychologic characterization of the elite distance runner. Ann. N. Y. Acad. Sci. 301, 382–403. 10.1111/j.1749-6632.1977.tb38215.x 270929

[B31] Nogueira A. Molinero O. Salguero A. Márquez S. (2018). Exercise addiction in practitioners of endurance sports: a literature review. Front. Psychol. 9, 1484–1484. 10.3389/fpsyg.2018.01484 30174636 PMC6107830

[B32] O'Connor P. J. Cook D. B. (1999). Exercise and pain: the neurobiology, measurement, and laboratory study of pain in relation to exercise in humans. Exerc. Sport Sci. Rev. 27, 119–166. 10.1249/00003677-199900270-00007 10791016

[B33] O'Connor P. J. Murphy R. M. Courson R. W. Ferrara M. S. (2000). Pain assessment in Journal of Athletic Training articles (1992–1998): Implications for improving research and practice. J. Athl. Train. 35, 151–154. 16558624 PMC1323411

[B34] Ouellette J.A. Wood W. (1998). Habit and intention in everyday life: The multiple processes by which past behavior predicts future behavior. Psych. Bull. 124, 54–74. 10.1037/0033-2909.124.1.54

[B35] Owen P. J. Miller C. T. Mundell N. L. Verswijveren S. J. Tagliaferri S. D. Brisby H.. (2019). Which specific modes of exercise training are most effective for treating low back pain? Network meta-analysis. Br. J. Sports Med. 54:1279. 10.1136/bjsports-2019-100886 31666220 PMC7588406

[B36] Rebar A. Dimmock J. A. Jackson B. Rhodes R. E. Kates A. Starling J. (2016). A systematic review of the effects of non-conscious regulatory processes in physical activity. Health Psychol. Rev. 10, 395–407. 10.1080/17437199.2016.1183505 27118430

[B37] Riley J. L. Robinson M. E. Wise E. A. Myers C. D. Fillingim R. B. (1998). Sex differences in the perception of noxious experimental stimuli: a meta-analysis. Pain 74, 181–187. 10.1016/S0304-3959(97)00199-1 9520232

[B38] Rollman G. B. Abdel-Shaheed J. Gillespie J. M. Jones K. S. (2004). Does past pain influence current pain: biological and psychosocial models of sex differences. Eur. J. Pain 8, 427–433. 10.1016/j.ejpain.2004.03.002 15324774

[B39] Rollman G. B. Lautenbacher S. (2001). Sex differences in musculoskeletal pain. Clin. J. Pain 17, 20–24. 10.1097/00002508-200103000-00004 11289085

[B40] Roy R. Thomas M. Makarenko T. M. (1989). Memories of pain: comparison of “worst pain ever” experienced by senior citizens and college students. Clin. J. Pain 5, 359–362. 10.1097/00002508-198912000-00014 2520426

[B41] Satterthwaite P. Norton R. Larmer P. Robinson E. (1999). Risk factors for injuries and other health problems sustained in a marathon. Br. J. Sports Med. 33, 22–26. 10.1136/bjsm.33.1.22 10027053 PMC1756136

[B42] Schmitt A. Wallat D. Stangier C. Martin J. A. Schlesinger-Irsch U. Boecker H. (2020). Effects of fitness level and exercise intensity on pain and mood responses. Eur. J. Pain 24, 568–579. 10.1002/ejp.1508 31738468

[B43] Shanely R. A. Nieman D. C. Zwetsloot K. A. Knab A. M. Imagita H. Luo B.. (2014). Evaluation of Rhodiola rosea supplementation on skeletal muscle damage and inflammation in runners following a competitive marathon. Brain Behav. Immun. 39, 204–210. 10.1016/j.bbi.2013.09.005 24055627

[B44] Slapšinskaite A. Hristovski R. Razon S. Balagué N. Tenenbaum G. (2017). Metastable pain-attention dynamics during incremental exhaustive exercise. Front. Psychol. 7:2054. 10.3389/fpsyg.2016.02054 28111563 PMC5216051

[B45] Slimmon D. Bennell K. Brukner P. (2002). Long-term outcome of fasciotomy with partial fasciectomy for chronic exertional compartment syndrome of the lower leg. Am. J. Sports Med. 30, 581–588. 10.1177/03635465020300041901 12130414

[B46] Sparling P. B. Nieman D. C. O'Connor P. J. (1993). Selected scientific aspects of marathon racing. Sports Med. 15, 116–132. 10.2165/00007256-199315020-00005 8446823

[B47] Staiano W. Bosio A. De Morree H. M. Rampinini E. Marcora S. (2018). The cardinal exercise stopper: muscle fatigue, muscle pain or perception of effort? Prog. Brain Res. 240, 175–200. 10.1016/bs.pbr.2018.09.012 30390830

[B48] Stevens C. J. Mauger A. R. Hassmèn P. Taylor L. (2018). Endurance performance is influenced by perceptions of pain and temperature: theory, applications and safety considerations. Sports Med. 48, 525–537. 10.1007/s40279-017-0852-6 29270865

[B49] Tokinoya K. Ishikura K. Ra S.-G. Ebina K. Miyakawa S. Ohmori H. (2020). Relationship between early-onset muscle soreness and indirect muscle damage markers and their dynamics after a full marathon. J. Exerc. Sci. Fitness 18, 115–121. 10.1016/j.jesf.2020.03.001 32351588 PMC7183207

[B50] Watson K. B. Frederick G. M. Harris C. D. Carlson S. A. Fulton J. E. (2015). U.S. adults' participation in specific activities: behavioral risk factor surveillance system-−2011. J. Phys. Activity Health 12, S3–S10. 10.1123/jpah.2013-0521 25157914 PMC4589138

