# Supplementary material for: Pain During a Marathon Run: Prevalence and Correlates in a Cross-Sectional Study of 1,251 Recreational Runners in 251 Marathons
Source: Front Sports Act Living. 2021 Feb 10;3:630584. doi: 10.3389/fspor.2021.630584 (PMC7902858; doi:10.3389/fspor.2021.630584)
Supplement: Supplementary file 1 [file Table_1.DOC]

**INFORMED CONSENT**

Please read the following information carefully. After reading this form, you will decide whether or not you wish to participate in the research described.

I am Pat O’Connor a researcher in the Department of Kinesiology at the University of Georgia.

I invite you to participate in a research study titled “Pain Experience in Marathon Runners”.

The purpose of the study is to learn about the pain that people experience when training for and running a 26.2 mile marathon.

Please do not participate if you are not 18 years old or over.

If you agree to be in this study, you will complete a web-based survey that asks about your experiences before, during and after your most recent 26.2 mile marathon run. It should take about 10 minutes to complete. You can skip any questions that you do not wish to answer. Once you have completed the survey, your answers can be sent over the Internet by clicking on the Submit button at the end of the questionnaire. If you click the Discard button, your responses will not be recorded.

Please be aware that Internet communications can be insecure. We cannot guarantee your privacy and confidentiality while the data are transmitted to us over the Internet. However, once we receive the completed surveys, any information that is obtained in connection with this study and that can be identified with you will remain confidential except as required by law. The results from your participation will be anonymous. A summary of the research results from all participants will be published but no mention will be made of individual results. Participants who are uncomfortable with the online questionnaire can print it out, complete it, and send it by regular mail to the researcher at the address provided below.

Your participation is voluntary. You may refuse to participate or discontinue participation at any time without penalty or loss of benefits to which you are otherwise entitled.

By participating, you will receive a 20% discount when purchasing running shoes, apparel or accessories from the online retailer www.runningwarehouse.com. Also, you will be entered for a chance to win a certificate worth $75 that can be applied toward the purchase of running shoes, apparel or accessories from the online retailer www.runningwarehouse.com. There will be 10 winners among the first 1,000 participants. Accordingly, you have a 1% chance of winning. If you have won, you will be informed immediately after completing the survey. Only one survey per computer will be accepted.

There are no known risks or discomforts associated with this research.

The principle researcher, Dr. Patrick J. O’Connor, will be happy to answer any questions about the questionnaire and research project and he can be reached using the contact information given below.

Additional questions or problems regarding your rights as a research participant should be addressed to The Chairperson, Institutional Review Board, University of Georgia, 612 Boyd Graduate Studies Research Center, Athens, Georgia 30602-7411; Telephone (706) 542-3199; E-Mail Address IRB@uga.edu

**QUESTIONS WERE PRESENTED IN ONLINE FORMAT**

1) What is your gender?

____Female

____Male

____I choose to not answer this question

2) What is your age?

____years

____I choose to not answer this question

3) What is your height?

____Inches or

____Centimeters

____I choose to not answer this question

4) What was the intensity of the most intense pain you have ever experienced?

____0 no pain at all

____½ very faint pain (just noticeable)

____1 weak pain

____2 mild pain

____3 moderate pain

____4 somewhat strong pain

____5 strong pain

____6

____7 very strong pain

____8

____9

____10 extremely intense pain (almost unbearable pain)

____ unbearable pain – if your pain was unbearable insert a number above 10 that is relative to 10

(example: 15 is 1.5 times as intense as 10 and 20 is twice as intense as 10)

____I choose to not answer this question

5) How many marathons have you run?

I have run ____ marathons

____I choose to not answer this question

6) What was the name of the most recent marathon you ran?

__________________marathon

____I choose to not answer this question

7) How many weeks or months has it been since you ran this most recent marathon?

____ weeks

____ months

____I choose to not answer this question

8) How many weeks or months did you train specifically for this most recent marathon?

____ weeks

____ months

____I choose to not answer this question

9) During weeks you trained for this most recent marathon, what was the average number of days per week that you trained?

____days

____I choose to not answer this question

10) During weeks you trained for this most recent marathon, what was the duration of your longest training runs

_____ minutes or

_____ hours

____I choose to not answer this question

11) During weeks you trained for this most recent marathon, what was the duration of your training runs on average

_____ minutes

____I choose to not answer this question

12) During weeks you trained for this most recent marathon, what was the overall, average intensity of your training runs?

____6

____7 Very, very light

____8

____9 Very light

____10

____11 Fairly light

____12

____13 Somewhat hard

____14

____ 15 Hard

____ 16

____ 17 Very hard

____ 18

____ 19 Very, very hard

____I choose to not answer this question

13) On what percentage of the training days for this most recent marathon did you experience running-induced pain?

____ zero percent of training days

____ percent (insert percent from 1 to 100)

____I choose to not answer this question

14) During those training days you experienced running-induced pain, what was the primary location of the pain?

___I was not experiencing any pain

____toe

____heel of foot

____arch of foot

____ball of foot

____top of foot

____achilles

____ankle

____shin

____calf

____lateral part of the knee

____medial part of the knee

____front part of the knee

____thigh

____hamstrings

____buttocks

____groin area

____hip

____hip flexors

____ iliotibial band

____low back

____upper back

____abdominal area

____chest area

____shoulders

____arms, hands and fingers

____neck

____head

____some other site

____I choose to not answer this question

15) During the training runs that you experienced running-induced pain, what was the overall average intensity of the pain felt at the primary pain location?

____0 no pain at all

____½ very faint pain (just noticeable)

____1 weak pain

____2 mild pain

____3 moderate pain

____4 somewhat strong pain

____5 strong pain

____6

____7 very strong pain

____8

____9

____10 extremely intense pain (almost unbearable pain)

____ unbearable pain – if your pain was unbearable insert a number above 10 that is relative to 10

(example: 15 is 1.5 times as intense as 10 and 20 is twice as intense as 10)

____I choose to not answer this question

16) How many hours of sleep do you usually get?

____ hours (to the quarter hour – 6.75 or 8.25)

____I choose to not answer this question

17) How many hours of sleep did you get the night before your most recent marathon?

____ hours (to the quarter hour – 6.75 or 8.25)

____I choose to not answer this question

18) What nutritional supplements did you take before the race on the day of your most recent marathon?

____ I did not take any supplement

____ name of supplement

____ dose of supplement

____I choose to not answer this question

19) What nutritional supplements did you take during the race on the day of your most recent marathon?

____ I did not take any supplement

____ name of supplement

____ dose of supplement

____I choose to not answer this question

20) What over-the-counter pain medicines did you take before the race on the day of your most recent marathon? Examples include – advil, alleve, aspirin, ibuprofen, motrin, and tylenol

____ I did not take any medicine

____ name of medicine

____ dose of medicine

____I choose to not answer this question

21) What over-the-counter pain medicines did you take during the race on the day of your most recent marathon? Examples include – advil, alleve, aspirin, ibuprofen, motrin, and tylenol

____ I did not take any medicine

____ name of medicine

____ dose of medicine

____I choose to not answer this question

22) What prescription medicines did you take before the race on the day of your most recent marathon?

____ I did not take any medicine

____ name of medicine

____ dose of medicine

____I choose to not answer this question

23) What caffeine containing pill, beverage or product did you take before the race on the day of your most recent marathon? Examples include – No-doze or Vivarin pills, coffee, tea, soda drinks such as Pepsi, Coca-Cola and Mountain Dew, energy drinks such as Red Bull, chocolate and hot cocoa.

____ I did not take any caffeine

____ type of substance (pill, beverage, food)

____ name of substance

____ amount of substance

____I choose to not answer this question

24) What military time of day did your most recent marathon start?

____ hours____minutes

____I choose to not answer this question

25) What was the approximate temperature at the start of your most recent marathon?

____Farenheit or

____Celsius

____I choose to not answer this question

26) Indicate the dominant wind conditions during your most recent marathon.

____No wind at all

____Light

____Moderate

____Strong

____I choose to not answer this question

27) What was your body weight at the start of the last marathon that you ran?

____Pounds or

____kilograms

____I choose to not answer this question

28) Resting at the starting line of your last marathon, what was the primary location of any pain you were experiencing?

___I was not experiencing any pain

____toe

____heel of foot

____arch of foot

____ball of foot

____top of foot

____achilles

____ankle

____shin

____calf

____lateral part of the knee

____medial part of the knee

____front part of the knee

____thigh

____hamstrings

____buttocks

____groin area

____hip

____hip flexors

____ iliotibial band

____low back

____upper back

____abdominal area

____chest area

____shoulders

____arms, hands and fingers

____neck

____head

____some other site

____I choose to not answer this question

29) Resting at the starting line of your last marathon, what was the intensity of pain you were experiencing?

____0 no pain at all

____½ very faint pain (just noticeable)

____1 weak pain

____2 mild pain

____3 moderate pain

____4 somewhat strong pain

____5 strong pain

____6

____7 very strong pain

____8

____9

____10 extremely intense pain (almost unbearable pain)

____ unbearable pain – if your pain was unbearable insert a number above 10 that is relative to 10

(example: 15 is 1.5 times as intense as 10 and 20 is twice as intense as 10)

____I choose to not answer this question

30) At what mile did you begin to feel pain during your last marathon?

At mile _____

_____ I choose to not answer this question

31) What was the primary location of the pain you experienced during your most recent marathon?

___I was not experiencing any pain

____toe

____heel of foot

____arch of foot

____ball of foot

____top of foot

____achilles

____ankle

____shin

____calf

____lateral part of the knee

____medial part of the knee

____front part of the knee

____thigh

____hamstrings

____buttocks

____groin area

____hip

____hip flexors

____ iliotibial band

____low back

____upper back

____abdominal area

____chest area

____shoulders

____arms, hands and fingers

____neck

____head

____some other site

____I choose to not answer this question

32) At the primary location of pain during your last marathon, what was the average intensity of pain you were experiencing? By pain we mean the intensity of hurt, separate from other feelings such as discomfort or fatigue.

____0 no pain at all

____½ very faint pain (just noticeable)

____1 weak pain

____2 mild pain

____3 moderate pain

____4 somewhat strong pain

____5 strong pain

____6

____7 very strong pain

____8

____9

____10 extremely intense pain (almost unbearable pain)

____ unbearable pain – if your pain was unbearable insert a number above 10 that is relative to 10

(example: 15 is 1.5 times as intense as 10 and 20 is twice as intense as 10)

____I choose to not answer this question

33) At the primary location of pain during your last marathon, what was the highest intensity of pain you were experiencing?

____0 no pain at all

____½ very faint pain (just noticeable)

____1 weak pain

____2 mild pain

____3 moderate pain

____4 somewhat strong pain

____5 strong pain

____6

____7 very strong pain

____8

____9

____10 extremely intense pain (almost unbearable pain)

____ unbearable pain – if your pain was unbearable insert a number above 10 that is relative to 10

(example: 15 is 1.5 times as intense as 10 and 20 is twice as intense as 10)

____I choose to not answer this question

34) What was the overall intensity of the effort you gave in your last marathon?

____6

____7 Very, very light

____8

____9 Very light

____10

____11 Fairly light

____12

____13 Somewhat hard

____14

____ 15 Hard

____ 16

____ 17 Very hard

____ 18

____ 19 Very, very hard

____I choose to not answer this question

35) What was your finishing time in your most recent marathon?

_____ I did not finish

My time was _____ hours and _____minutes and _____ seconds

____I choose to not answer this question

36) How many hours of sleep did you get the night after your most recent marathon?

____ hours (to the quarter hour – 6.75 or 8.25)

____I choose to not answer this question

37) What was the primary location of the pain you experienced during the 1 to 3 days after your most recent marathon?

___I was not experiencing any pain

____toe

____heel of foot

____arch of foot

____ball of foot

____top of foot

____achilles

____ankle

____shin

____calf

____lateral part of the knee

____medial part of the knee

____front part of the knee

____thigh

____hamstrings

____buttocks

____groin area

____hip

____hip flexors

____ iliotibial band

____low back

____upper back

____abdominal area

____chest area

____shoulders

____arms, hands and fingers

____neck

____head

____some other site

____I choose to not answer this question

38) At the primary location of pain you felt on days 1-3 after your last marathon, what was the highest intensity of pain you experienced?

____0 no pain at all

____½ very faint pain (just noticeable)

____1 weak pain

____2 mild pain

____3 moderate pain

____4 somewhat strong pain

____5 strong pain

____6

____7 very strong pain

____8

____9

____10 extremely intense pain (almost unbearable pain)

____ unbearable pain – if your pain was unbearable insert a number above 10 that is relative to 10

(example: 15 is 1.5 times as intense as 10 and 20 is twice as intense as 10)

____I choose to not answer this question

39) Check all the body locations that were painful during the 1 to 3 days after your most recent marathon?

___I was not experiencing any pain

____toe

____heel of foot

____arch of foot

____ball of foot

____top of foot

____achilles

____ankle

____shin

____calf

____lateral part of the knee

____medial part of the knee

____front part of the knee

____thigh

____hamstrings

____buttocks

____groin area

____hip

____hip flexors

____ iliotibial band

____low back

____upper back

____abdominal area

____chest area

____shoulders

____arms, hands and fingers

____neck

____head

____some other site

____I choose to not answer this question

40) How many consecutive days after the marathon did you experience pain that was caused by the marathon?

____ I had no days of pain

I had _____ consecutive days of pain

____I choose to not answer this question

41) How did you treat any pain you were experiencing in the days after the marathon

____I did not treat pain

____I rested

____I took an over-the-counter pain medicine

____I took a prescription medicine

____I stretched

____I was massaged

____I applied heat

____I applied cold

____I had chiropractic care

____Other
